# Identifying bedrest using 24-h waist or wrist accelerometry in adults

**DOI:** 10.1371/journal.pone.0194461

**Published:** 2018-03-23

**Authors:** J. Dustin Tracy, Sari Acra, Kong Y. Chen, Maciej S. Buchowski

**Affiliations:** 1 Energy Balance Laboratory, Division of Gastroenterology, Hepatology and Nutrition, Department of Medicine, Vanderbilt University Medical Center, Nashville, Tennessee, United States of America; 2 Department of Pediatrics, Vanderbilt University Medical Center, Nashville, Tennessee, United States of America; 3 National Institute of Diabetes and Digestive and Kidney Diseases, Diabetes, Endocrinology, and Obesity Branch, National Institutes of Health, Bethesda, Maryland, United States of America; University of Georgia, UNITED STATES

## Abstract

**Objectives:**

To adapt and refine a previously-developed youth-specific algorithm to identify bedrest for use in adults. The algorithm is based on using an automated decision tree (DT) analysis of accelerometry data.

**Design:**

Healthy adults (n = 141, 85 females, 19–69 years-old) wore accelerometers on the waist, with a subset also wearing accelerometers on the dominant wrist (n = 45). Participants spent ≈24-h in a whole-room indirect calorimeter equipped with a force-platform floor to detect movement.

**Methods:**

Minute-by-minute data from recordings of waist-worn or wrist-worn accelerometers were used to identify *bedrest* and *wake* periods. Participants were randomly allocated to development (n = 69 and 23) and validation (n = 72 and 22) groups for waist-worn and wrist-worn accelerometers, respectively. The optimized DT algorithm parameters were *block length*, *threshold*, *bedrest-start trigger*, and *bedrest-end trigger*. Differences between DT classification and synchronized objective classification by the room calorimeter to *bedrest* or *wake* were assessed for sensitivity, specificity, and accuracy using a Receiver Operating Characteristic (ROC) procedure applied to 1-min epochs (n = 92,543 waist; n = 30,653 wrist).

**Results:**

The optimal algorithm parameter values for *block length* were 60 and 45 min, *thresholds* 12.5 and 400 counts/min, *bedrest-start trigger* 120 and 400 counts/min, and *bedrest-end trigger* 1,200 and 1,500 counts/min, for the waist and wrist-worn accelerometers, respectively. *Bedrest* was identified correctly in the validation group with sensitivities of 0.819 and 0.912, specificities of 0.966 and 0.923, and accuracies of 0.755 and 0.859 by the waist and wrist-worn accelerometer, respectively. The DT algorithm identified *bedrest/sleep* with greater accuracy than a commonly used automated algorithm (Cole-Kripke) for wrist-worn accelerometers (p<0.001).

**Conclusions:**

The adapted DT accurately identifies bedrest in data from accelerometers worn by adults on either the wrist or waist. The automated bedrest/sleep detection DT algorithm for both youth and adults is openly accessible as a package “PhysActBedRest” for the R-computer language.

## Introduction

Accelerometry-based technology for health and wellness tracking is expanding rapidly, outpacing the ability to validate the data generated and creating a barrier to employing these devices in clinical and research settings, which might otherwise benefit from the rich data provided[1,2]. Wearable accelerometers have become a major tool for the measurement of physical activity (PA), the prediction of PA-induced energy expenditure, and sleep assessment[3,4]. Although the detailed analysis of human sleep requires polysomnography (PSG) measures, accelerometry is considered a reasonably reliable and valid alternative method to estimate sleep-wake patterns[3,5]

Technological advances such as watch-like waterproof devices with large data storage capacity allow assessing PA for extended monitoring periods (e.g., 24 hours/day for seven days)[6]. This “24/7” approach has gained gradual acceptance in research because it improves the ability to examine associations between physical activity, sedentary behaviors, sleep, and health in the natural or free-living environment[6]. Accelerometers for PA assessment have been commonly worn on the waist or hip, but a moderate compliance rate in participants for wearing these devices demonstrated by free-living studies has led to the use of wrist-worn accelerometers, especially for assessing sleep patterns in cross-sectional and epidemiological studies[6,7].

Analysis of the 24-h per day and multiple-day accelerometer recordings from free-living requires a comprehensive approach. This includes assessing adherence to the monitor-wearing protocol using a wearing/nonwearing algorithm or other methodologies[8,9]. The next step is to discriminate periods of sleep or bedtime rest periods from wake periods encompassing sedentary behaviors as well as more active periods commonly categorized as light, moderate, and vigorous intensity PA. Especially challenging is distinguishing nighttime sleep and daytime naps from sedentary behaviors[10].

Traditionally, sleep periods under free-living conditions have been assessed using self-reports, or more objectively, recordings from accelerometers equipped with a light sensor, an inclinometer, or an event button[11]. An alternative approach is to use automated algorithms that classify accelerometer wear-time into the bedrest/sleep and wake periods using empirically determined cut points from the accelerometer output (i.e., counts) such as those developed for wrist-worn accelerometers in children and adults by Sadeh or Cole-Kripke, respectively[12,13]. Although these algorithms were specifically developed to identify wake periods during a time in bed or sleep, they are commonly used as automated algorithms to detect sleep in 24-h accelerometer data[14]. Similar algorithms based on accelerometry recordings or body posture classification to identify sleep in young adults and children have been developed[4,15–18]. The major concern about the validity of sleep-wake scoring algorithms is their relatively low specificity defined as an ability to identify wake intervals correctly during sleep period [19].

We previously developed a decision tree (DT) to identify the time in bedrest within 24-h data collected using Actigraph accelerometers worn by healthy youth ages 10–18 years on either their waist or wrist[20]. Although the algorithm showed good accuracy to separate bedrest from wake in the youth population, its validity cannot be assumed for adults with different personal characteristics and irregular bedtime habits. Thus, the primary goal of this study was to adapt this DT to identify bedrest periods in adults and compare the results with objective classification by a whole-room indirect calorimeter. The performance of the DT for a wrist-worn accelerometer was compared to the Cole-Kripke automated algorithm[13] labeling of *Sleep* and *Awake* obtained using a proprietary program (ActiLife v. 6.13.3, Actigraph, Pensacola, FL, USA) to analyze Actigraph data[21]. Our secondary goal was to integrate algorithm parameters into a DT capable of identifying bedrest in both youth and adult data and making this algorithm openly accessible.

## Methods

### Study participants

Healthy adult volunteers (n = 141, 19 to 69 years old) were recruited from Nashville, Tennessee in the USA using flyers, emails, and word-of-mouth for a prospective study focused on PA assessment methodology in adults[22]. All applicable institutional and governmental regulations concerning the ethical use of human volunteers were followed in accordance with the ethical principles of the Helsinki-II Declaration. The study protocol and consent form were approved by the Institutional Review Board of the Vanderbilt University (Approval Number: 040293). All participants signed an informed consent before the study. Study data were collected from 2006 to 2009, and current analyses were performed in 2017.

### Study design and protocol

Study participants spent ≈24-h in a whole-room indirect calorimeter where they followed a protocol designed to simultaneously measure PA and energy expenditure with high precision in near-naturalistic conditions, as described previously[23]. The force-platform covering the floor inside the calorimeter allowed measurement (60 times/second) of overall body position, displacement, and mechanical forces with 97% or higher accuracy[23]. All (n = 141) participants wore Actigraph accelerometer on their dominant side waist, and some participants (n = 45) wore an Actigraph on their dominant wrist.

The daytime PA protocol was designed to simulate free-living PA patterns found in population studies. The protocol included: a) sedentary behaviors (≈40%) such as sitting and viewing TV/media, b) light intensity PA (≈50%) such as eating meals, gaming, performing personal care, and unscripted normal daily routines, c) moderate PA (≈8%) such as walking and jogging on a treadmill, and d) vigorous PA (≈2%) such as running on a treadmill and biking. Participants were instructed to start bedrest around 10:00 pm and they were prompted to wake up at 6:00 am. Anticipating participants might depart from protocol, bedrest was defined as the time spent on a mattress bed when the force platform detected no significant movement and energy expenditure was at or below resting energy expenditure[20]. Wake was defined as time spent off the mattress when the force platform detected movement and energy expenditure was higher than resting energy expenditure. An interruption in bedrest was defined as a period equal or longer than 5 minutes (5 consecutive 1-min epochs) classified by the room calorimeter as *wake*.

PA was measured using Actigraph GT1M uniaxial accelerometer (ActiGraph, Pensacola, FL) that generates data in counts per user-defined time sampling intervals (i.e., epochs) manufacturer-provided firmware (v. 6.2.0) and software (ActiLife v. 6.13.3. In this study, accelerometry recordings were collected at 1-sec epoch and reintegrated as counts per minute to synchronize with data from the room calorimeter (energy expenditure) and force platform (mechanical work). The lag time between Actigraph recordings and the room calorimeter data (90 seconds) was accounted for in the analyses.

### Measures

#### Movement-induced mechanical work (Watt/min)

Movement-induced horizontal and vertical mechanical work (Watt/min) was measured using the force platform sensitive to small pressure changes caused by a participant’s movement[24].

#### Energy expenditure (kcal/min)

Minute-by-minute energy expenditure (kcal/min) was calculated from measured rates of O_2_ consumption and CO_2_ production using Weir’s equation[23]. The accuracy of our room calorimeter for measuring energy expenditure has been previously documented[23].

#### Room calorimeter classified sleep/rest and wake

Room calorimeter-measured energy expenditure and the force platform-measured mechanical work threshold values and plots were used to classify 1-min epochs as a *sleep/rest* or a *wake* binary indicator variable and were synchronized minute-by-minute with the accelerometer recordings data, as described previously[20].

### Development of a decision tree for bedrest and wake classification

To identify bedrest and wake epochs from accelerometer recordings, we adapted an automated DT we had previously developed for youth[20] by testing various combinations of the selected algorithm parameters values. The parameters were *block length*, *threshold*, *bedrest-end trigger*, and *bedrest-start trigger*. The *block length* was defined as the number of epochs over which an average number of counts per epoch was calculated; this effectively generates a set period (e.g., if a block had 60 epochs and epoch was 1-min, then *block length* was 60-min). The *threshold* was the value (counts/min) for which block averages falling below or rising above were assumed to represent a transition from *wake* to *bedrest* or from *bedrest* to *wake*. The *bedrest-end trigger* was a minimum number of counts/min allowed in any two consecutive 1-min epochs to be marked as bedrest end. The next epoch was the start of *wake*. The *bedrest-start trigger* was the minimum number of counts/min required in any two consecutive 1-min epochs to be marked as wake end. The next epoch was the start of bedrest.

**Fig 1:**
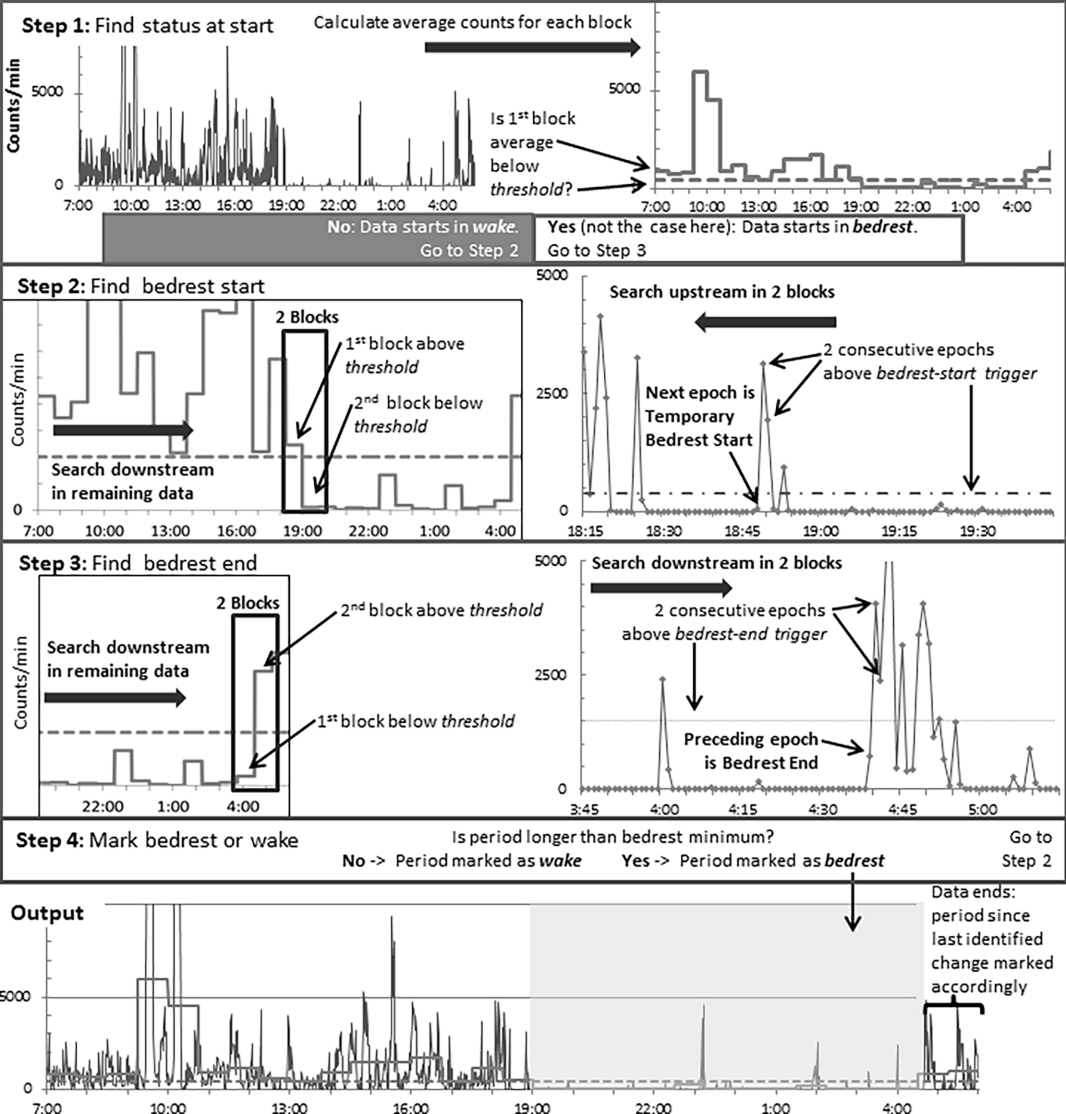
Simplified decision tree (DT) for the classification of accelerometer recordings (counts/epoch) as *bedrest* or *wake*. The DT uses different algorithm parameters values (*block length*, *threshold*, *bedrest-end trigger*, *and bedrest-start trigger*) for waist-worn and wrist-worn accelerometers and has a four-step process to cycle through the data.

The DT has four steps presented in Fig 1. In Step 1, DT divides the entire accelerometer recording dataset (e.g., 24-h) into time blocks, (e.g., 60-min), calculates the average counts per epoch for each block (e.g., counts/minute), and compares it to the *threshold*. If the 1^st^ block average is equal to or higher than the *threshold*, the 1^st^ epoch is marked as *wake* and DT proceeds to Step 2. If the 1^st^ block average is less than the *threshold*, the 1^st^ epoch of this block is marked as a temporary bedrest-start and DT proceeds to Step 3.

In Step 2, DT identifies bedrest-start. It searches for a 2-block window in which the 1^st^ block’s average is equal or higher and the 2^nd^ block’s average lower than the *threshold*. After finding such window, DT searches upstream data in the window or a 2-epoch interval with the number of counts/min higher than *bedrest-start trigger*.- If such interval is found, the 1^st^ epoch that follows the interval is marked as a *temporary bedrest-start*. Otherwise, the 1^st^ epoch of the 2-block window is marked as temporary bedrest-start. Epochs preceding the temporary bedrest start are marked as *wake*.

In Step 3, DT identifies bedrest-end. It searches for a 2-block window in which the 1^st^ block average is lower, and the 2^nd^ block average is equal to or higher than the *threshold*. After finding such window, DT searches downstream in data in the window for a 2-epoch interval with the number of counts/min higher than *bedrest-end trigger*. If such interval is found, the epoch before the interval is marked as a temporary bedrest end and following epochs are classified as *wake*.

In Step 4, DT classifies each temporary bedrest period as *bedrest* or *wake*. If the temporary bedrest period is shorter than a specified minimum bedrest period (30 min for waist and 60 min for wrist), the temporary bedrest-start and bedrest-end are discarded and the period is marked as *wake*. If the temporary bedrest period is equal or longer than a minimum bedrest period, it is marked as *bedrest*. The next epoch is marked as *wake*, and DT repeats Step 2 with the remainder of the dataset. If in either Step 2 or Step 3, DT reaches the last epoch in the dataset; all epochs from the last identified change from *bedrest* to *wak*e or *wake* to *bedrest* until the dataset end are marked accordingly as *wake* or *bedrest*. The detailed DT description is in S1 Appendix and S1 Fig.

The DT’s assumption that *bedrest* periods have a minimum length potentially results in some short naps being falsely labeled as *wake*. However, it does guard against waking periods of low activity (e.g., sedentary behavior) being falsely labeled as *bedrest*. For both waist and wrist, we set the minimum bedrest length to 0, 30, and 60 minutes, and report parameters that maximized the accuracy score. The R function allows the user to set the minimum bedrest period to balance these concerns.

### Data analysis

The participants were assigned to development and validation groups separately for the wrist- and wrist-worn accelerometers using a list-randomizer available at *random*.*or*g. The development group was used to identify the optimal combination of algorithm parameters values (*block length*, *threshold*, *start and end triggers)*. An automated program constructed and tested trial combinations. For each combination and each participant, every epoch (1-min) in the monitoring period (≈24-h) was classified by DT as *bedrest* or *wake* and compared to time-synchronized *sleep/rest* or *wake* classification from the room calorimeter. Each epoch (n = 92,543 waist; n = 30,653 wrist) was then compared to the corresponding room calorimeter classification and categorized as true positive, true negative, false positive or false negative[25].

The ROC-curves were plotted with each point representing the sensitivity and specificity in identifying epochs as *bedrest* or *wake* of a trial combination[26]. Sensitivity was defined as the probability of correctly identifying *bedrest* (accelerometry = *bedrest* when room-calorimetry = *sleep/rest*), and specificity was defined as the probability of correctly classifying *wake* (accelerometry = *wake* when room-calorimetry = *wake*). Specificity and sensitivity were considered equally important. For each combination of *block lengths* (0, 30, and 60 min), *threshold* (from 7.5 to 500 counts/min), *bedrest-start trigger* (from 100 to 2,000 counts/min), and *bedrest-end trigger* (from 100 to 400 counts/min), medians of sensitivity, specificity, and accuracy (sensitivity*specificity) were calculated for the development group. The optimal algorithm parameters values obtained from the development group were tested using the validation group. The 2-fold validation method was chosen based on a sufficient sample size in the development (n = 69 and 23) and validation sets (n = 72 and 22) for waist-worn and wrist-worn accelerometers, respectively. The differences in accuracy of selected algorithm parameters values were tested using the Wilcoxon signed-rank test.

To assess performance of DT, we compared its accuracy for the validation group to the results obtained from a wrist-worn Actigraph using automated Cole-Kripke sleep detection algorithm[13] in ActiLife software[21]. As we had done with DT, the Cole-Kripke results (*Sleep* or *Awake*) were synchronized minute-by-minute and compared with the room calorimeter’s classifications (*sleep/rest* or *awake*).

### Statistical analysis

Data from waist- and wrist-worn accelerometer recordings (counts/min) were analyzed separately. Results are presented as means or medians, standard deviations (SD), and ranges. For the optimal algorithm parameters values, the differences in accuracy between the development and validation groups were tested using the Wilcoxon signed rank test. The DT *bedrest* and Cole-Kripke algorithm *Sleep* classifications for wrist-worn accelerometer were compared using the Wilcoxon signed rank test since the outcome distributions were skewed. Linear regression was used to test if differences in accuracy between development and validation sets were correlated with race, age, sex, or BMI separately for waist- and wrist-worn accelerometer groups. The programming language R version 2.15.2[27] was used to develop DT. Statistical significance was set at p < 0.05 and Stata software (Release 13, Stata Corp. 2013, College Station, TX, USA) was used to perform statistical analyses.

## Results

### Participants’ characteristics

There were no significant differences (all *p* > 0.05) in personal characteristics between participants in the development and validation groups for both waist-worn and wrist-worn monitors (Table 1). Although the protocol suggested the bedtime be from 10 pm to 6 am, the actual bedrest/sleep patterns varied substantially which presented classification challenges similar to those expected in free living. Characteristics of bedrest in both groups are presented in Table 2. Room calorimeter classified sleep/bedrest length among study participants varied from less than 3 hours to more than 11 hours of the ≈24-h room calorimeter stay. A number of interruptions in bedrest defined as a period longer than equal or longer than 5 min varied from 0 to 6. On average, participants had interruptions that totaled 18.8 min and 18.1 min in the waist- and wrist-worn accelerometer groups, respectively. A number of sleep episodes starting and ending before 10 pm ranged from 0 to 5 and totaled on average 53.1 min and 33.2 min in the waist- and wrist-worn accelerometer groups, respectively.

**Table 1:** Characteristics of study participants.

Waist-worn accelerometer^a^					Wrist-worn accelerometer^a^			
	All participants(n = 141)	Development Group (n = 69)	Validation Group(n = 72)	*p*^b^	All participants(n = 45)	Development Group(n = 23)	Validation Group (n = 22)	*p*^b^
Age (years)	39.7± 13.6 (19–69)	38.9±13.9(20–69)	40.5 ± 13.3(19–67)	0.49	40.3 ±13.9(20–67)	42.1±16.1(20–67)	38.8 ± 11.3 (20–59)	0.44
Height (m)	1.69 ± 0.10(1.52–1.91)	1.70± 0.10 (1.52–1.89)	1.69 ± 0.09 (1.54–1.91)	0.43	1.67 ± 0.09 (1.16–1.84)	1.67 ± 0.09 (1.54–1.83)	1.67 ± 0.09(1.55–1.84)	0.84
Weight (kg)	77.8 ± 19.1 (47.8–134.5)	77.4 ± 17.8(47.8–123.5)	78.3± 20.3 (48.7–134.5)	0.79	84.1 ± 21.2(47.8–130.2)	79.3 ± 19.4 (47.8–123.5)	89.2 ± 21.8(58.5–130.2)	0.25
BMI^c^ (kg/m^2^)	27.2 ± 6.6 (16.9–52.0)	26.8 ± 5.9 (16.9–52.1)	27.6 ± 7.1(17.9–51.3)	0.49	29.9 ± 7.9(19.3–52.1)	28.5 ± 7.3(19.3–52.1)	31.5 ± 8.2(22.3–51.3)	0.33
*Sex*								
Female	85	39	46		29	13	16	
Male	56	30	26		16	10	6	
*Ethnicity*								
Black	43	19	24		31	13	18	
White	95	48	47		13	9	4	
Other	3	2	1		1	1	0	

^a—^values are presented as mean ± standard deviation and (range)

^b^- two-sample t-test

^c^- BMI—body mass index (body weight [kg]/ height [m^2^].

**Table 2:** Characteristics of *bedrest* and *wake* periods.

	Waist-worn accelerometer^a^(n = 141)	Wrist-worn accelerometer^a^(n = 45)
Bedrest (min)	480.8 ± 78.6(178–700)	469.1 ± 90.1(178–646)
Wake (min)	864 ± 81.4(638–1164)	867.7 ± 92.6(700–1164)
Interruptions in bedrest after 10 pm	0.81 ± 0.89(0–6)	0.56 ± 0.61(0–2)
Time of interruptions (min)	18.63 ± 27.95(10–174)	17.93 ± 26.90(10–118)
Number of bedrest incidents ending before 10 pm or after 6 am (min)	1.26 ± 1.25(0–5)	0.75 ± 0.68(0–3)
Total time of bedrest incidents ending before 10 pm or after 6 am (min)	51.03 ± 58.71(10–299)	33.16 ± 36.44(10–125)

^a—^values are presented as mean ± standard deviation and (range).

### Decision tree algorithm parameters

The most accurate combinations of algorithm parameters values tested are in Table 3. A larger dataset is in S1 Table.

**Table 3:** Medians for accuracy, sensitivity, and specificity for selected combinations of algorithm parameters. The development group medians are reported for Receiver Operating Characteristic (ROC) procedures for waist-worn and wrist-worn accelerometry data. Optimal combinations are shown in bold.

Threshold (counts/min)	Bedrest end Trigger (counts/min)	Bedrest start trigger (counts/min)	Block length (min)	Accuracy SD^a^			Sensitivity^b^	Specificity^c^
**Waist**								
10	1200	120	60	0.761		0.150	0.821	0.968
10	1200	130	60	0.761		0.146	0.831	0.965
12.5	1100	120	60	0.767		0.146	0.851	0.954
12.5	1100	130	60	0.767		0.144	0.852	0.952
12.5	1200	110	60	0.767		0.148	0.842	0.954
12.5	1200	120	30	0.688		0.129	0.916	0.800
12.5	1200	120	45	0.731		0.138	0.880	0.868
12.5	1200	120	60	0.774		0.147	0.851	0.954
12.5	1200	130	30	0.686		0.129	0.916	0.800
12.5	1200	130	45	0.731		0.141	0.880	0.868
**12.5**	**1200**	**130**	**60**	**0.774**		**0.145**	**0.852**	**0.952**
12.5	1200	140	60	0.767		0.143	0.852	0.952
12.5	1300	120	60	0.774		0.147	0.851	0.954
12.5	1300	130	60	0.774		0.145	0.852	0.952
12.5	1400	120	60	0.774		0.148	0.851	0.954
12.5	1400	130	60	0.774		0.146	0.852	0.952
12.5	1500	120	60	0.774		0.148	0.851	0.954
12.5	1500	130	60	0.774		0.146	0.852	0.952
15	1200	120	60	0.763		0.158	0.857	0.948
15	1200	130	60	0.763		0.155	0.862	0.941
**Wrist**								
200	500	300	45	0.899	±	0.091	0.910	0.993
250	1000	300	30	0.896	±	0.065	0.961	0.978
250	1500	400	45	0.896	±	0.065	0.927	0.990
350	1250	350	45	0.893	±	0.057	0.953	0.974
400	1000	250	45	0.896	±	0.054	0.963	0.968
400	1000	400	45	0.896	±	0.061	0.969	0.968
**400**^**d**^	**1500**	**400**	**45**	**0.892**	**±**	**0.063**	**0.969**	**0.961**
400	1750	300	45	0.890	±	0.056	0.966	0.931
400	2000	250	60	0.894	±	0.080	0.922	0.979
450	1000	350	30	0.893	±	0.060	0.966	0.918
450	1250	350	45	0.879	±	0.063	0.966	0.931
450	1500	350	60	0.884	±	0.072	0.934	0.988

a—calculated as sensitivity multiplied by specificity before results were rounded

^b^—the probability of correctly classifying *bedrest;*

^c^—the probability of correctly classifying *wake*

^**d**^—optimal combination (bolded).

#### Optimal time block and threshold

The optimal *block length* for the threshold average searching was 60-min for waist-worn and 45-min for wrist-worn accelerometer. The selected *threshold* was 12.5 counts/min for the waist and 400 counts/min for the wrist accelerometer.

#### Bedrest end triggers and start triggers

The selected values for *bedrest-end trigger* were 1,200 and 1,500 counts/min and for *bedrest-start trigger* were 120 and 400 counts/min for the waist- and wrist-worn accelerometer, respectively.

#### Comparison between development and validation sets

For the waist accelerometer, accuracy (0.774 and 0.755) did not differ between the development and validation datasets (*p* = 0.606). For the wrist accelerometer, accuracy (0.896 and 0.859) differed between the development and validation datasets (p = 0.019) (Table 4).

**Table 4:** Comparison of medians of *bedres*t classification from waist- or wrist-worn accelerometer in the development and validation groups with classification obtained using room calorimeter.

Monitor placement	Group	Sensitivity^b^	Specificity^c^	Accuracy^a^	*p*^d^
Waist	Development (n = 69)^e^^,^	0.852	0.952	0.774	0. 606
	Validation (n = 72)^e^	0.819	0.966	0.755	
Wrist	Development (n = 23)^e^^,^ ^f^	0.969	0.968	0.896	0.019
	Validation (n = 22)^f^	0.912	0.923	0.859	

^a^—calculated as sensitivity multiplied by specificity before results were rounded

^b^—the probability of correctly classifying *bedrest*

^c^—the probability of correctly classifying *wake*

d—Wilcoxon signed rank test

e—optimal *block length* was 60 min, *threshold* 12.5 counts/min, *bedrest-start trigger* 120 counts/min, and *bedrest-end trigger* 1,200 counts/min

f—optimal *block length* was 45 min, *threshold* 400 counts/min, *bedrest-start trigger* 400 counts/min, and *bedrest-end trigger* 1,500 counts/min.

**Fig 2:**
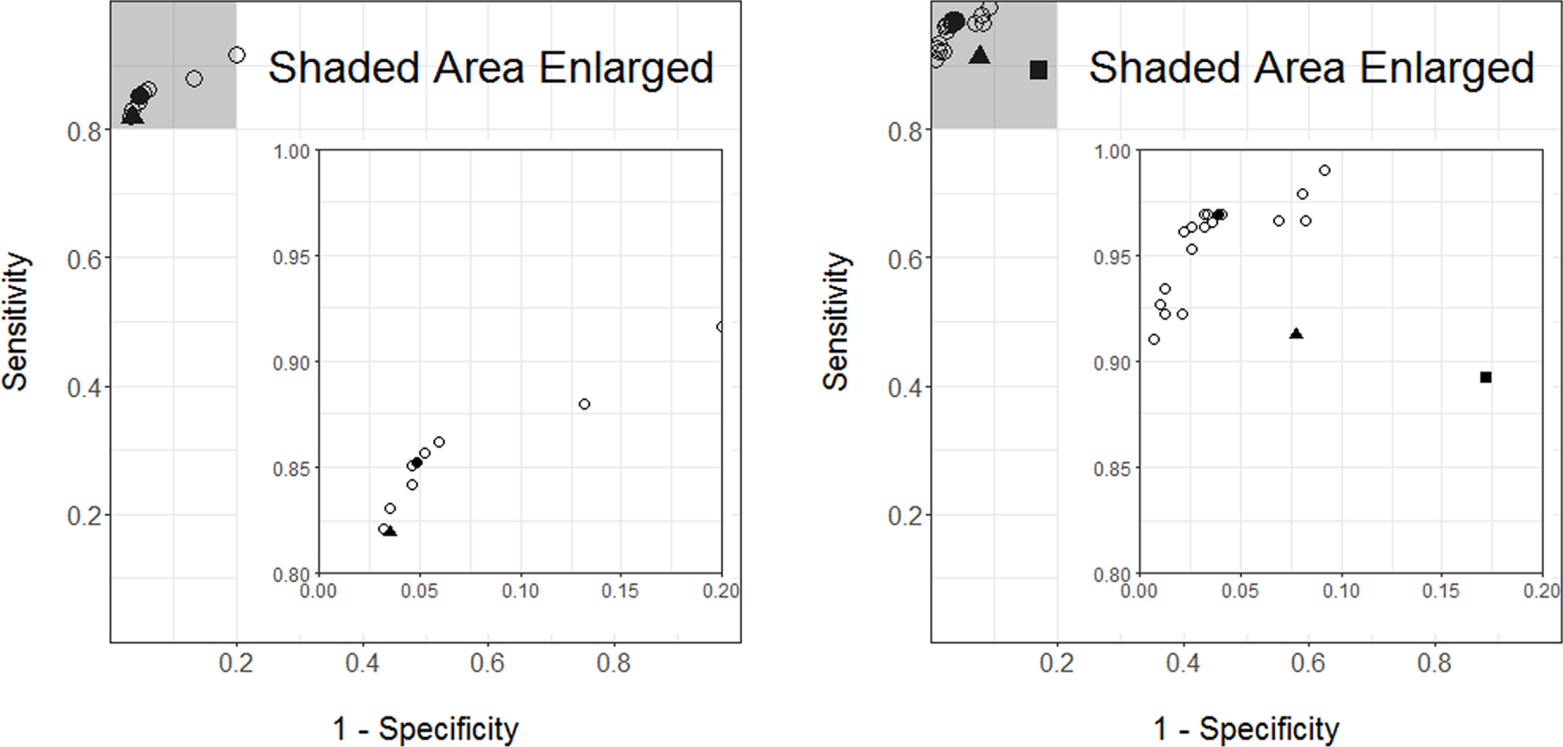
Plots of showing the tradeoff between sensitivity (y-axis) and 1-specificity (x-axis). (A) Data from waist-worn accelerometers (B) Data from wrist-worn accelerometers. Each open circle [○] represents a respective combination of *threshold* (counts/min) for *bedrest end* (counts/min), *bedrest start* (counts/min), and *block length* (min). The solid circle [●] represents the selected optimal combination. The corresponding values are in Table 2 (bolded). The solid triangle [▲] represents the validation set. The solid square [■] in B represents Cole-Kripke algorithm.

The accuracy in classification to *bedrest* or *wake* between the development and validation datasets was not associated with race, gender, age, and BMI in wrist-worn group and race, gender, and BMI in waist-worn accelerometer group (all *p* >0.05) (S2 Table). The accuracy of classification to *bedrest* or *wake* was higher for the wrist than waist accelerometer (*p* <0.001). The ROC curves plotted using the medians of sensitivity and 1-specificity are in Fig 2, for waist (A) and wrist (B) accelerometers respectively.

#### Comparison of DT and automated Cole-Kripke algorithm bedrest classification

Our DT with optimized algorithms parameters identified *bedrest* improved over the automated Cole-Kripke algorithm adopted for wrist-worn Actigraph data [21] in the validation group regarding sensitivity (0.912 and 0.891), specificity (0.923 and 0.828), and accuracy (0.859 and 0.763) (all *p*<0.001; Table 5).

**Table 5:** Comparison of medians of *bedrest* classification from accelerometer placed on wrist calculated using Cole-Kripke automated algorithm and the decision tree (DT) with classification obtained using room calorimeter.

Bedrest assessment method	Sensitivity^a^	Specificity^b^	Accuracy^c^	*p*^d^
Algorithm (Cole-Kripke)^e^	0. 907	0. 806	0.711	<0.019
Decision tree (DT)^f^	0.912	0.923	0.859	

^a^—the probability of correctly classifying *bedrest*

^b^—the probability of correctly classifying *wake*

^c^—calculated as sensitivity multiplied by specificity before results were rounded

d—Wilcoxon signed rank test

e—available in the proprietary software (ActiLife v. 6.13.3, Actigraph, Pensacola, FL, USA) to analyze Actigraph data

f—optimal *block length* was 45 min, *threshold was* 400 counts/min, *bedrest-start trigger* was 400 counts/min, and *bedrest-end trigger* was 1,500 counts/min.

## Discussion

In this study, DT that we previously developed to identify bedrest periods using waist- or wrist-worn accelerometers recordings for youth[20] was adapted for use in adults. The adapted DT provides good (>0.75) sensitivity and specificity to identify *bedrest* and *wake*.

The need for an accurate methodology to differentiate *bedrest* (sleep) from *wake* periods is growing because of recent advances that have allowed wearing relatively nonintrusive movement monitors for several days. For example, the National Health and Nutrition Survey (NHANES) study is currently assessing PA using a wrist-worn accelerometer worn 24-h per day for seven days[28]. Separating bedrest that includes sleep from sedentary behaviors and PA intensity categories in ≈24-h per day accelerometer recordings is necessary for continuous monitoring, unlike in “wake hours” protocols in which participants are asked to wear a monitor only from “waking up until going to bed, except during water-related activities”[6].

In this study, we did not attempt to assess physiological sleep but rather to identify periods of inactivity as *bedrest*, which most likely included sleep and longer daytime naps exceeding 60 minutes. We used the terms *bedrest* and *wake* to define periods below or above the optimal *threshold* at which *bedrest* was identified. In the current accelerometry literature, terms used for similarly defined “inactivity” range from “sleep” and “sleep-period time” to “nocturnal sleep” and “in-bed time”[17,29,30]. The measurement of physiological sleep requires polysomnography which is considered as a gold standard when investigating sleep patterns [31]. However, the method is expensive, time-consuming, and difficult to carry out in free-living individuals. In recent years, several studies have shown that accelerometry is ≈80 to 90% concordant with polysomnography during night rest in adults and children[16,32,33]. Although accelerometry does not provide the detailed information on sleep that polysomnography does, it has the advantages of portability, tolerability, and the possibility to identify sleep patterns and quantify between-day sleep variability in free-living[3]. According to the Society of Behavioral Sleep Medicine (SBSM), actigraphy can provide useful information for sleep clinicians about the patient's sleep at home over several nights as well as change in sleep over time and thus, inform clinical decision making[34].

The optimal algorithm parameters for adults differed from values we have established for youth[20], underscoring a need for population-specific values for accurate identification of *bedrest* and *wake*. The variation was caused, at least in part, by differences in movement patterns of adults (18 to 65 years old) compared to those of youth (10 to 18 years old). Although the study protocols in the room calorimeter were very similar in both studies, the process by which we searched for optimal values had slight differences. When establishing optimal criteria for youth, we set *block length* at 60 min and *bedrest start trigger* at 50 counts/min. In the current modification of DT, these values are allowed to vary affecting *bedrest end trigger* and *threshold*. In addition, we tested a broader range of values for adults than for youth, so the recommended values for youth might represent a local rather than global maximum. Future research will revisit calibration for youth with this improved methodology. Finally, there was greater heterogeneity of movement (counts/min) during bedrest in adults compared to youth. For example, intra-individual differences expressed as a mean standard deviation of movement between 10:00 pm and 6:00 am was 273 and 124 counts/min for waist-worn and 1035 and 488 counts/min for wrist-worn accelerometers for adults and youth, respectively (S3 Table).

The sensitivity and especially specificity of waist and wrist classification were lower than in our previous study in youth[20]. Similarly, in past studies, specificity defined as ability to correctly identify wake was also higher in children and adolescents than in adults [32,35]. It is very likely that the intra-individual differences in total amount and variability of movement during bedrest and wake among the participants decreased both specificity and sensitivity as did extra-individual differences between the groups. For example, standard deviation of means (counts/min) during the ≈24-h stay was higher in adults than in youth (167 and 227 counts/min and 53 and 178 counts/min) for waist-worn and wrist-worn accelerometers, respectively (S3 Table).

We assessed DT efficiency compared to an automated Cole-Kripke sleep scoring algorithm for adults[13] developed for wrist-worn accelerometers and specifically; for assessing sleep during bedtime but commonly used for comparison with other automatic algorithms[36]. Relative to the room calorimeter classification, the Cole-Kripke algorithm had lower sensitivity and specificity resulting in lower accuracy than our DT. A plausible explanation could be that the Cole-Kripke algorithm classified short (e.g., 1-min) inactivity episodes during *wake* (daytime) as “Sleep.” In contrast, in DT *bedrest* periods were limited to inactivity lasting at least 30 min for waist and 60-min for wrist-worn accelerometer. It is possible that imposing this limitation could have caused misclassification of some short periods with very low intensity (e.g., daytime naps) as *wake*.

We did not directly compare our DT to other available automatic algorithms, since they were developed and validated in different populations[17,30], used different methodology[7], or different accelerometers[11]. Among algorithms validated for waist-worn Actigraph accelerometers, one of the first was an algorithm developed by Tudor-Locke et al.[29] for children combining visual inspection used to mark onset and offset of sleep from Actigraph data with sleep diary. The newer version of this algorithm showed a moderately high correlation of nocturnal sleep (r = 0.61 to 0.74) with sleep diaries and visual assessment of accelerometry data[30].

Recently, McVeigh and colleagues[17] validated an automated algorithm to separate “in-bed time” from “waking” data in young adults using visual inspection of accelerometry data collected from waist-worn Actigraph as a reference method. The median sensitivity of their algorithm was higher (0.95 and 0.82) and median specificity comparable (0.95 and 0.97) to our waist-worn accelerometer data. The differences between the studies might be caused, at least in part, by differences in participants’ characteristics (e.g., age, BMI) and study environment.

In this study, we found the accuracy of *bedrest* and *wake* classification was higher for wrist than waist-worn accelerometer. Similarly, Slater et al.[37] and Zikham at al.[16] showed that relative to the waist, a wrist-worn Actigraph GTX3+ provided a more valid assessment of polysomnography-measured sleep. In addition to the documented higher adherence to wearing wrist versus waist monitor [38], this finding might support the use of wrist accelerometers for 24-h monitoring of bedrest in free-living studies.

Our study had several strengths. The room-calorimeter allowed us to classify *sleep/rest* and *wake* using objective minute-by-minute measurements of energy expenditure and mechanical work for ≈24-h. Utilizing recordings from accelerometers placed on waist and wrist allowed us to compare sensitivity, specificity, and accuracy between these common monitor placements[39]. Random selection of the development and validation sets allowed a robust performance of the algorithm. We used a relatively large (n = 141) and diverse group regarding of sex, race, age, and body mass indices (S2 Table). Time spent in bedrest varied from 3 to 11 hours, which is similar to sleep time range in general USA adult population[40,41].

The study had some limitations. First, it was conducted under laboratory conditions that minimized measurement error but limited the DT generalizability. Thus, one should expect some loss of accuracy when extending out to free-living conditions with more variability in the underlying sets of behaviors than in this study. The DT was developed in a study lasting ≈24-h with bedrest following a relatively active period of wearing. However, the variability in bedrest time among study participants very likely offset, at least in part, this limitation. Nonetheless, longer monitoring with other scenarios regarding wake and bedrest that would normally occur with free-living individuals was not examined. Thus, we advise users to apply the wearing-nonwearing algorithm[8] before applying the current DT and interpret the results with caution.

Second, we used a uniaxial accelerometer, since supplanted by triaxial models. However, it has been shown that uniaxial and triaxial accelerometers were comparable when assessing rest and sedentary behaviors[42]. Utilizing recordings from one axis is a conventional approach to studies assessing sleep using accelerometry[16,17] since it enables cross-study comparisons. Besides, the Cole-Kripke automated algorithm we used to assess performance of our DT uses recordings from the same Actigraph axis we utilized. Nevertheless, DT should be recalibrated using different accelerometry measures (e.g., vector magnitude) and recordings from accelerometers collecting raw data. Third, our study population was restricted to healthy adults aged 19 to 68 years old and not reporting sleep disorders. It is likely that sleep and activity patterns in older populations and those with sleep disorders might require different model parameters values[43]. Finally, although we did not test the differences in the model parameters values selection between males and females or black and white adults, such differences may emerge in larger studies. Future research should test our DT in diverse populations, calibrate it against polysomnography, and compare with other automatic algorithms using recordings from accelerometers worn continuously for several days [6].

In summary, we adopted an automated decision tree (DT) originally developed to identify bedrest periods in ≈24-h accelerometer count recordings from waist-worn or wrist-worn accelerometer worn by youth to adults in the current study. The parameters optimized in DT were *block length*, *threshold*, *bedrest-end trigger*, and *bedrest-start trigger*. The adapted DT provided good (>0.75) sensitivity and specificity to identify *bedrest* and *wake* and identified bedrest with higher accuracy from wrist-worn than waist–worn accelerometers in adults. The optimal values for the DT parameters selected using ROC procedure were different from the values we have established for youth underscoring a need for population-specific values for accurate identification of *bedrest* and *wake*. The automated DT allows replacing the default algorithm parameters values with values specified by a user.

## Conclusions

Accelerometry data collected from wrist- or waist-worn monitors for 24-h can be used to accurately identify bedrest apart from sedentary behaviors and activity in adults. The automated bedrest/sleep detection DT algorithm for both youth and adults is openly accessible as a package “PhysActBedRest” for the R-computer language.

## Supporting information

S1 Table Tested bedrest and wake thresholds for the waist-worn and waist-worn accelerometers. (XLSX)

S2 Table Relationship of gender, race, age, and BMI with error in detecting bedrest between development and validation sets in waist and wrist-worn accelerometer groups. (DOCX)

S3 Table Intraindividual and extraindividual differences in the amount of movement (counts) in adults and youth. (DOCX)

S1 Fig The decision tree (DT) for the classification of accelerometer recordings (counts/epoch) as *bedrest* or *activity*. (TIF)

S1 Appendix Decision tree (DT) development. (DOCX)
