# Supplementary material for: Identifying bedrest using 24-h waist or wrist accelerometry in adults
Source: PLoS One. 2018 Mar 23;13(3):e0194461. doi: 10.1371/journal.pone.0194461 (PMC5865746; doi:10.1371/journal.pone.0194461)
Supplement: S2 Table — (DOCX) [file pone.0194461.s002.docx]

S2 Table. Relationship of gender, race, age, and BMI with error in detecting bedrest between development and validation sets in waist and wrist-worn accelerometer groups.

| **Variable** | **Coefficient** | **Standard error** | ***p* value** | **95% CI^a^** | |
| --- | --- | --- | --- | --- | --- |
| Waist (n=141) | | | | | |
| Gender^b^ | 0. 0535 | 0.0370 | 0. 150 | -0. 0196 | 0.127 |
| Age [years] | -0.00266 | 0.000933 | 0.00867 | -0.0126 | 0.0106 |
| Race^c^ | -0.00628 | 0. 0291 | 0. 830 | -0.0638 | 0.0513 |
| Weight | -0. 000980 | 0.00586 | 0.867 | -0. 0126 | 0. 0106 |
| Height | 0. 172 | 0.605 | 0.777 | -1.026 | 1. 369 |
| BMI^d^ | 0. 000128 | 0. 0163 | 0. 994 | -0. 0321 | 0. 0324 |
| Wrist (n=45) | | | | | |
| Gender^b^ | -0.0500 | 0.0501 | 0.324 | -0.1511 | 0.0514 |
| Age [years] | -0.000535 | 0.00112 | 0.636 | -0.00174 | 0.00281 |
| Race^c^ | -0.0453 | 0.0330 | 0.178 | -0.112 | 0.0215 |
| Weight | 0.00222 | 0.00717 | 0.759 | -0.0123 | 0.0167 |
| Height | -0.413 | 0.799 | 0.609 | -2.030 | 1.205 |
| BMI^d^ | 0.000260 | 0.00200 | 0.977 | -0.00656 | 0.00136 |

^a^- Confidence Intervals

^b^ - 0 - male, 1 - female

^c^ - 0 -White, 1 - Black or Hispanic

^d^ - BMI - body mass index (body weight [kg]/ height [m^2^]

e - R-squared = 0.214

^f^ - R-squared = 0.085
