# Supplementary material for: Identifying bedrest using 24-h waist or wrist accelerometry in adults
Source: PLoS One. 2018 Mar 23;13(3):e0194461. doi: 10.1371/journal.pone.0194461 (PMC5865746; doi:10.1371/journal.pone.0194461)
Supplement: S3 Table — (DOCX) [file pone.0194461.s003.docx]

S3 Table. Intraindividual and extraindividual differences in the amount of movement (counts) in adults and youth^.^

|  | Adults^a^ | | Youth^b^ | |
| --- | --- | --- | --- | --- |
|  | Waist (n=141) | Wrist (n=45) | Waist (n=49) | Wrist (n=49) |
| Night (10 pm -6 am) |  |  |  |  |
| Mean_i(SD_t (counts)^c^ | 123.64 | 1034.98 | 272.55 | 488.31 |
| Entire Period (≈24-h stay) |  |  |  |  |
| SD_i(Mean_t(counts))^d^ | 167.43 | 226.80 | 53.02 | 178.19 |

^a -^ Data are from the present study (adults). Raw data are on figshare <https://doi.org/10.6084/m9.figshare.5959957>

^b -^ Data from previous study in youth (reference # 22). Raw data are on figshare <https://doi.org/10.6084/m9.figshare.5965408>

^c –^ Data were collected minute by minute for all participants. For each participant, the standard deviation of counts in the period from 10 pm to 6 am was calculated. The mean across participants of individual SDs for each participant are reported. Mean_i(SD_t (counts)).

^d -^ Data were collected minute by minute for all participants. For each participant, the mean of counts during the entire stay was calculated. The standard deviation across participants of individual means for each participant are reported. SD_i(Mean_t(counts)).
