# Supplementary figures and images for: Identifying bedrest using 24-h waist or wrist accelerometry in adults
Source: PLoS One. 2018 Mar 23;13(3):e0194461. doi: 10.1371/journal.pone.0194461 (PMC5865746; doi:10.1371/journal.pone.0194461)

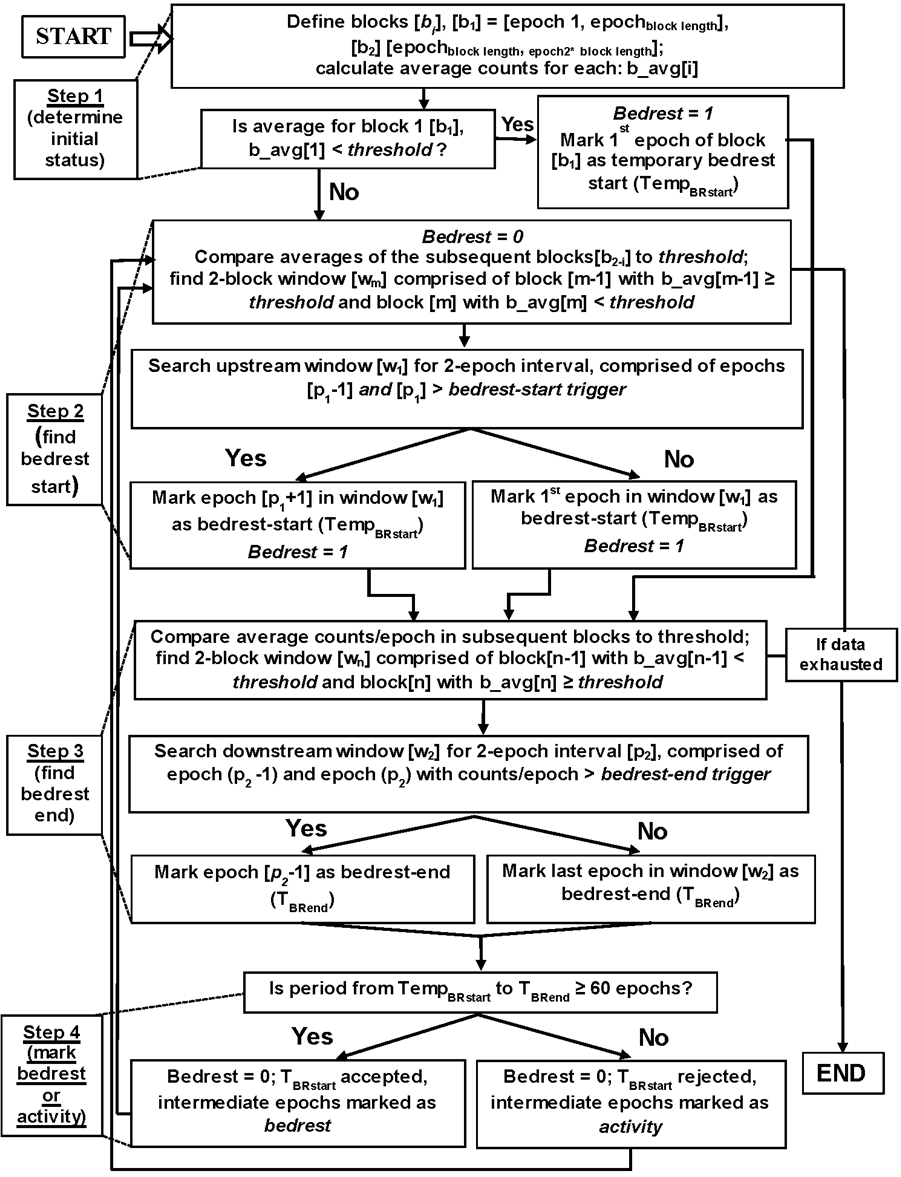

Supplement: S1 Fig — (TIF) [file pone.0194461.s004.tif]
