# Supplementary material for: Identifying bedrest using 24-h waist or wrist accelerometry in adults
Source: PLoS One. 2018 Mar 23;13(3):e0194461. doi: 10.1371/journal.pone.0194461 (PMC5865746; doi:10.1371/journal.pone.0194461)
Supplement: S1 Appendix — (DOCX) [file pone.0194461.s005.docx]

**Appendix to manuscript by Tracy et al.** "Identifying bedrest using 24-h waist or wrist accelerometry in adults.”

**Appendix 1: Decision Tree (DT) Development**

DT was developed to identify epochs (e.g., 1 minute) in accelerometry data (e.g., counts/minute) as *bedrest* or *activity* using following predetermined criterion variables:

- ***block length***: number of epochs (e.g., minutes) for which average number of counts per epoch is calculated (e.g., if a block has 60 epochs and epoch is 1 minute, then *block length* is 60 minutes)
- ***threshold***: optimal number of counts per epoch (e.g. counts/minute) for comparing with the average counts per epoch within a block when searching for transition time points from *bedrest* to *activity* and *activity* to *bedrest.*
- ***bedrest-end trigger****:* minimum number of counts per epoch (e.g., counts/minute) allowed in any two consecutive epochs to be marked as start of *activity*
- ***bedrest-start trigger***: minimum number of counts per epoch (e.g., counts/minute) required in any two consecutive epochs to be marked as end of *activity*

Other variables and their definitions:

- epoch: period of time in which accelerometry recordings are rendered (e.g., minute)
- block*:* cluster of epochs (e.g., minutes) used for calculating average number of counts per epoch (e.g. counts/minute)
- temporary bedrest-start: 1^st^ epoch of the block in which average counts/epoch (e.g., 200 counts/min) is less than *threshold* (e.g., 500 counts/min)
- window: cluster of epochs comprised of two blocks (e.g., 2 blocks * 60-min/block =120 minutes) in which change from *bedrest* to *activity* or *activity* to *bedrest* occurs.
- bedrest-start: epoch within a window in which the change from *activity* to *bedrest* occurs
- bedrest-end: epoch within a window in which the change from to *bedrest* to *activity* occurs
- minimum bedrest period: number of epochs in a period (e.g., 60 min) to be classified as bedrest.

DT has four Steps.

In Step 1, DT divides the entire accelerometer recordings dataset (e.g., 24 hours) into time blocks, each being *block length* epochs long (e.g., 60 minutes), and then calculates the average counts per epoch (e.g. counts/minute) for each block. If the 1^st^ block average is less than the *threshold,* the 1^st^ epoch of this block is marked as a temporary bedrest-start and DT proceeds to Step 3. If the 1^st^ block average is equal to or higher than *threshold*, the 1^st^ epoch of this block is marked as *activity* and DT proceeds to Step 2.

In Steps 2 and 3, DT marks bedrest-end and bedrest-start epochs for each bedrest period within each block.  DT identifies these events, by initially searching the blocks averages to identify a window in which the change occurred and then searches epoch-by-epoch through this window to detect the epoch in which the change occurred.

In Step 2, DT compares the blocks averages calculated in Step 1 to *threshold* and searches for a two-block window (e.g. 2 blocks * 60 min [*block length*] = 120 min) in which the 1^st^-block average is equal or higher than *threshold* and the 2^nd^ block average is lower than *threshold*.  After finding such window, DT searches upstream (starting from the last epoch) through it for a two-epoch interval with the number of counts/epoch higher than *bedrest-start trigger*.  If such interval is found, 1^st^ epoch that follows it is marked as a temporary bedrest-start.  If no such, interval is found, the 1^st^ epoch of the window is marked as temporary bedrest-start, and DT proceeds to Step 3 to process remaining blocks in the dataset.

In Step 3, DT compares the average counts/epoch in subsequent blocks to *threshold* until it finds a two-block window (e.g. 120 minutes) in which the 1^st^ block average (counts/epoch) is lower than *threshold* and the 2^nd^ block average is equal to or higher than *threshold*.  When such window is identified, DT searches it downstream until it finds a two-epoch interval with counts/epoch higher than *bedrest-end trigger*. If no such interval is found, the last epoch of the window is marked as bedrest-end. If such interval is found, the epoch before the 1^st^ epoch of this interval is marked as a temporary bedrest-end.

In Step 4, DT examines the length of the temporary bedrest period.  If the period is shorter than minimum bedrest period (e.g., 60 epochs), the temporary bedrest-start mark is discarded, and all epochs in this period are marked as *activity*.  If the period is equal or longer than minimum bedrest period (e.g., 60 minutes), all epochs in this period are marked as *bedrest*.  The next epoch is marked as *activity*, and DT repeats Step 2 with the remaining blocks in the dataset.

If DT, either in Step 2 or Step 3, reaches the last epoch in the dataset (dataset end), all epochs from the last identified change are marked according to the change (e.g. if a bedrest-end was the last identified change, all remaining epochs in the dataset are marked as *activity*).

**S1 Fig:** The decision tree (DT) for the classification of accelerometer recordings (counts/epoch) as *bedrest* or *activity*. The DT uses different criterion variable (*block length, threshold, bedrest-end trigger, and bedrest-start trigger*) values for waist and wrist worn accelerometers.
